# Genomic mining of *Vibrio parahaemolyticus* highlights prevalence of antimicrobial resistance genes and new genetic markers associated with AHPND and *tdh* + */trh* + genotypes

**DOI:** 10.1186/s12864-024-10093-9

**Published:** 2024-02-14

**Authors:** Marieke Vandeputte, Sieglinde Coppens, Peter Bossier, Nick Vereecke, Daisy Vanrompay

**Affiliations:** 1 Laboratory of Immunology and Animal Biotechnology, Department of Animal Production and Aquatic Ecology, Faculty of Bioscience Engineering, Ghent University, Ghent, Belgium; 2 Laboratory of Aquaculture & Artemia Reference Center, Department of Animal Production and Aquatic Ecology, Faculty of Bioscience Engineering, Ghent University, Ghent, Belgium; 3 PathoSense BV, Lier, Belgium

**Keywords:** *Vibrio parahaemolyticus*, Acute Hepatopancreatic Necrosis Disease, Whole genome sequencing, Antimicrobial resistance, Genome-wide association, Aquaculture, Shrimp, PirAB toxins

## Abstract

**Background:**

Acute Hepatopancreatic Necrosis Disease (AHPND) causes significant mortality in shrimp aquaculture. The infection is primarily instigated by *Vibrio parahaemolyticus* (*Vp*) strains carrying a plasmid encoding the binary toxin PirAB. Yet, comprehension of supplementary virulence factors associated with this relatively recent disease remains limited. Furthermore, the same holds for gastroenteritis in humans caused by other *Vp* genotypes. Additionally, given the prevalent use of antibiotics to combat bacterial infections, it becomes imperative to illuminate the presence of antimicrobial resistance genes within these bacteria.

**Results:**

A subsampled number of 1,036 *Vp* genomes was screened for the presence of antimicrobial resistance genes, revealing an average prevalence of 5 ± 2 (SD) genes. Additional phenotypic antimicrobial susceptibility testing of three *Vp* strains (M0904, TW01, and PV1) sequenced in this study demonstrated resistance to ampicillin by all tested strains. Additionally, *Vp* M0904 showed multidrug resistance (against ampicillin, tetracycline, and trimethoprim-sulfamethoxazole). With a focus on AHPND, a screening of all *Vibrio* spp. for the presence of *pirA* and/or *pirB* indicates an estimated prevalence of 0.6%, including four *V. campbellii*, four *V. owensii*, and a *Vibrio* sp. next to *Vp*. Their *pirAB*-encoding plasmids exhibited a highly conserved backbone, with variations primarily in the region of the Tn3 family transposase. Furthermore, an assessment of the subsampled *Vp* genomes for the presence of known virulence factors showed a correlation between the presence of the Type 3 Secretion System 2 and *tdh*, while the presence of the Type 6 Secretion System 1 was clade dependent. Furthermore, a genome-wide association study (GWAS) unveiled (new) genes associated with *pirA*, *pirB*, *tdh,* and *trh* genotypes. Notable associations with the *pirAB* genotype included outer membrane proteins, immunoglobulin-like domain containing proteins, and toxin-antitoxin systems. For the *tdh* + /*trh* + genotypes (containing *tdh, trh,* or both genes), associations were found with T3SS2 genes, urease-related genes and nickel-transport system genes, and genes involved in a ‘minimal’ type I-F CRISPR mechanism.

**Conclusions:**

This study highlights the prevalence of antimicrobial resistance and virulence genes in *Vp*, identifying novel genetic markers associated with AHPND and *tdh* + */trh* + genotypes. These findings contribute valuable insights into the genomic basis of these genotypes, with implications for shrimp aquaculture and food safety.

**Supplementary Information:**

The online version contains supplementary material available at 10.1186/s12864-024-10093-9.

## Background

Acute Hepatopancreatic Necrosis Disease (AHPND), previously referred to as Early Mortality Syndrome (EMS), is an emerging bacterial disease in shrimp. It has inflicted substantial economic losses on the shrimp industry, particularly in Asian countries like Thailand, Vietnam, and Malaysia, as well as in South America and the United States [1–4]. AHPND is caused by specific strains of the Gram-negative bacterium *Vibrio parahaemolyticus* carrying a conjugative plasmid (pVA1) of approximately 69 kbp in size, housing the *pirAB*^*vp*^ genes [5]. The binary pore-forming toxin PirAB is homologous to toxins secreted by *Photorhabdus* spp. (Pir) and is responsible for the characteristic lesions observed in the shrimp’s hepatopancreas [4, 5]. More recently, other *Vibrio* (*V.*) species have been reported to carry plasmids homologous to pVA1, that carry the *pirAB* genes, such as *V. harveyi* [6], *V. campbellii* [7–9], *V. owensii* [10] and *V. punensis* [11]. The plasmid-borne nature of these toxins facilitates their mobility and spread to different *Vibrio* species, which makes it more difficult to control the disease [12, 13]. Increased horizontal gene transfer of this mobile genetic element (MGE) is facilitated by the number of genes associated with conjugative transfer and plasmid mobilization, rendering the plasmid self-transmissible [1, 5]. This principle has been demonstrated in vitro by the successful horizontal transfer of a pVA1-type plasmid from *V. parahaemolyticus* to a non-pathogenic *V. campbellii* [14]. Furthermore, the plasmid encompasses a post-segregational killing system, ensuring its consistent inheritance [1, 5, 12, 15]. The plasmid contains a set of transfer-related genes, including the Tn903 (3.5 kbp) transposon where the *pirAB*^*vp*^ genes are located, and four transposases (ORF15, 48, 55, and 68) able to trigger the transfer of the DNA transposon [12, 16]. This has resulted in plasmid variants such as deletions of the *pirA* gene, total loss of *pirA,* and partial loss of *pirB* coding sequences [15, 17, 18].

Recent research has shown a geographical distinction between plasmids originating from Asian strains and Latin American strains. Specifically, Asian strains lack the Tn3 transposon that is present in all Latin American strains [19, 20]. However, a broader phylogenomic analysis by Yang et al*. (*2019) has suggested that *V. parahaemolyticus* is divided into four diverse populations, VppUS1, VppUS2, VppX, and VppAsia [21]. The VppUS1 and VppUS2 populations are largely restricted to the US and Northern Europe, while VppX and VppAsia are found worldwide, with VppAsia making up the great majority of seawater isolates around Asia [21].

Next to the aforementioned plasmid-encoded toxins, *V. parahaemolyticus* can contain chromosome-encoded virulence factors, such as hemolysins (thermostable direct hemolysin (TDH) and TDH-related hemolysin (TRH)), two Type III secretion systems (T3SS1 and T3SS2) and two T6SSs (T6SS1 and T6SS2). Genotypes carrying *tdh* and/or *trh* genes are highly pathogenic in humans, causing gastroenteritis after consumption of contaminated seafood. Throughout this manuscript, these genotypes are referred to as ‘*tdh* + */trh* + genotypes’. Additionally, T3SS2 was shown to be present in these genotypes. TDH, TRH, and T3SS2 are exclusively found in these human clinical isolates responsible for human acute gastroenteritis and are not related to pathogenesis in shrimp [22, 23]. In contrast, T3SS1 and T6SS2 are highly conserved and are suggested to play a role in virulence in both human infections and AHPND infections in shrimp. However, their working mechanisms are not fully understood yet [24, 25].

Over the last few years, aquaculture has expanded significantly, resulting in an increase in disease prevalence. This, in turn, has led to increased antibiotic usage among farms worldwide [26]. However, the improper, empirical, and extensive application of antibiotics has resulted in the emergence of antimicrobial resistance in a substantial amount of *V. parahaemolyticus* strains in the environment [27–29]. Every year, there is a reported increase in the multidrug resistance of pathogenic *V. parahaemolyticus* to clinically important antimicrobial drugs. This not only reduces the effectiveness of antibiotics used in aquaculture practices but also poses a threat to human health due to the transmission of *V. parahaemolyticus* strains carrying (mobilizable) antimicrobial resistance genes (ARGs) to humans upon consumption [27, 30, 31].

In the scope of this study, three distinct AHPND-associated *V. parahaemolyticus* strains were sequenced, originating from three different geographical sources: Mexico, Thailand, and China. The identification of antimicrobial resistance genes (ARGs) was linked to phenotypic antimicrobial susceptibility testing against a panel of 13 different antibiotics. In addition, using publicly available whole genome sequencing (WGS) data, extensive genomic mining, phylogenetics, and genome-wide association studies (GWAS) were performed to elucidate and characterize putative genetic mediators in pathogenic *V. parahaemolyticus* strains with a focus on AHPND and *tdh* + /*trh* + genotypes.

## Results

### Long-read whole genome sequencing statistics and quality check

As summarized in Additional file 1: Table S1, our newly sequenced isolates *Vp* M0904 (CP133891-CP133899), *Vp* PV1 (JAVKPG000000000), and *Vp* TW01 (CP133900-CP133905) showed 100% genome completeness, an average GC content of 45.3%, genome sizes of 5.6 Mbp (± 0.2 Mbp), longest contigs of 3.5 Mbp (± 0.04 Mbp), and an average of 5,718 (± 169) predicted genes. These values are all in line with the RIMD 2210633 *V. parahaemolyticus* (NC_004603.1) NCBI reference strain.

### Phenotypic antibiotic susceptibility testing and genomic ARG screening

The results from the antibiotic susceptibility testing can be found in Table 1. All three strains were resistant to ampicillin, with *Vp* M0904 showing additional resistance to piperacillin, tetracycline, and trimethoprim-sulfamethoxazole. Furthermore, both *Vp* TW01 and *Vp* PV1 were intermediately resistant to piperacillin. Interestingly, these phenotypes were supported by chromosome-encoded ARGs. While strains M0904 and PV1 encoded a *bla*_*CARB-23*_ beta-lactamase, strain *Vp* TW01 had a *bla*_*CARB-21*_ gene, explaining the observed phenotypes against ampicillin and piperacillin. Comparable, the *Vp* M0904 strain was the only strain carrying a *tet(B)* and *dfrA6* gene, supporting the resistance phenotypes against tetracycline and trimethoprim-sulfamethoxazole, respectively. Also, a *QnrVC5* gene was found in its genome, though no phenotypic resistance against any of the tested fluoroquinolones was observed. As shown in Additional file 2 and Additional file 1: Table S4, the *CRP*, *msbA*, *tet(35)*, and *ugd* genes were found in all three genomes.

**Table 1:** Results of antibiotic susceptibility tests of the *V. parahaemolyticus* (*Vp*) strains. Strains were resistant (R) or susceptible (S) to the antibiotics or showed an intermediate (I) profile. Identified ARGs, supporting the observed resistance phenotypes are included. A visual overview of all identified ARGS is given in Additional file 2

Antibiotic class	Antibiotic	*Vp* M0904	*Vp* TW01	*Vp* PV1
Β-lactam				
- Penams	ampicillin (10 µg)	R *bla*_*CARB-23*_	R *bla*_*CARB-21*_	R *bla*_*CARB-23*_
	amoxicillin-clavulanate (20/10 µg)	S	S	S
	ampicillin-sulbactam (10/10 µg)	S	S	S
	piperacillin (100 µg)	R *bla*_*CARB-23*_	I *bla*_*CARB-21*_	I *bla*_*CARB-23*_
- Cephalosporins	cefotaxime (30 µg)	S	S	S
	ceftazidime (30 µg)	S	S	S
Aminoglycosides	amikacin (30 µg)	S	S	S
	gentamicin (10 µg)	S	S	S
Tetracyclines	tetracycline (30 µg)	R *tet(B)*	S	S
Fluoroquinolones	ciprofloxacin (5 µg)	S	S	S
	levofloxacin (5 µg)	S	S	S
	ofloxacin (5 µg)	S	S	S
Folate pathway inhibitors	trimethoprim-sulfamethoxazole (5 µg)	R *dfrA6*	S	S

Whenever expanding our ARG screening to our subsampled dataset, we observed an average presence of 5 ± 2 (SD) ARGs per *V. parahaemolyticus* genome (Additional file 2 and Additional file 1: Table S4). This also confirmed the overall high prevalence of the *CRP* (99.7%), *msbA* (99.0%), *tet(35)* (99.3%), and *ugd* (84.4%) genes within the *V. parahaemolyticus* species. Notably, these genes showed relatively low nucleotide identity scores to the references as compared to other ARGs; 80.0% ± 0.1%, 66.5% ± 0.2%, 82.6% ± 0.2%, and 69.7% ± 0.9% nucleotide identity, for *CRP*, *msbA*, *tet(35)*, and *ugd*, respectively. A substantial amount of *V. parahaemolyticus* strains harbored either the *bla*_*CARB-23*_ (44.3%) or *bla*_*CARB-21*_ (55.1%) gene, suggesting a high resistance prevalence against β-lactams such as ampicillin and piperacillin. Other β-lactamases that were identified included the *bla*_*CARB-22*_ (0.2%), *bla*_*CTX-M-14*_ (3.1%), *bla*_*CTX-M-15*_ (0.1%), *bla*_*NDM-1*_ ( 0.2%), *bla*_*TEM-1*_ (0.1%), *bla*_*TEM-116*_ (0.1%), *bla*_*VEB-1*_ (2.2%). Aversely, resistance towards tetracycline and trimethoprim-sulfamethoxazole is suggested to be rather low as the *tet(B)* and *dfrA6* genes were only found in 3.1% and 4.1% of subsampled strains. Additionally, other tetracycline (*tet(59)* (1.9%), *tet(A)* (0.6%), *tet(C)* (0.1%), *tetI* (0.2%), and *tetM* (0.8%)) and trimethoprim-sulfamethoxazole-associated ARGs (*dfrA1* (0.1%), *dfrA16* (0.1%), *dfrA23* (0.1%), *dffrA27* (0.2%), *dfrA32* (0.1%), *sul1* (1.1%), and *sul2* (3.8%)) were identified. Of note, while a variety of other ARGs were identified at low prevalence (Additional file 1: Table S4), ARGs associated with aminoglycoside resistance ranged from 0.1% up to 3.1% prevalence, including the AAC(3)-IV, AAC(6’)-Iia, AAC(6’)-Ib9, ANT(2’’)-Ia, APH(3’’)-Ib, APH(3’)-Ia, APH(6)-Id. These genes seemed to be limited to strains showing a substantially higher (≥ 10 per strain) number of encoded ARGs. Furthermore, as shown in Additional file 2, this data is suggestive of the acquisition of a putative multidrug resistance MGE (*e.g.,* plasmid or integron) that defines a subclade within the VppAsia clade.

### Prevalence of AHPND-positive *Vibrio* species and associated plasmids

**Fig. 1:**
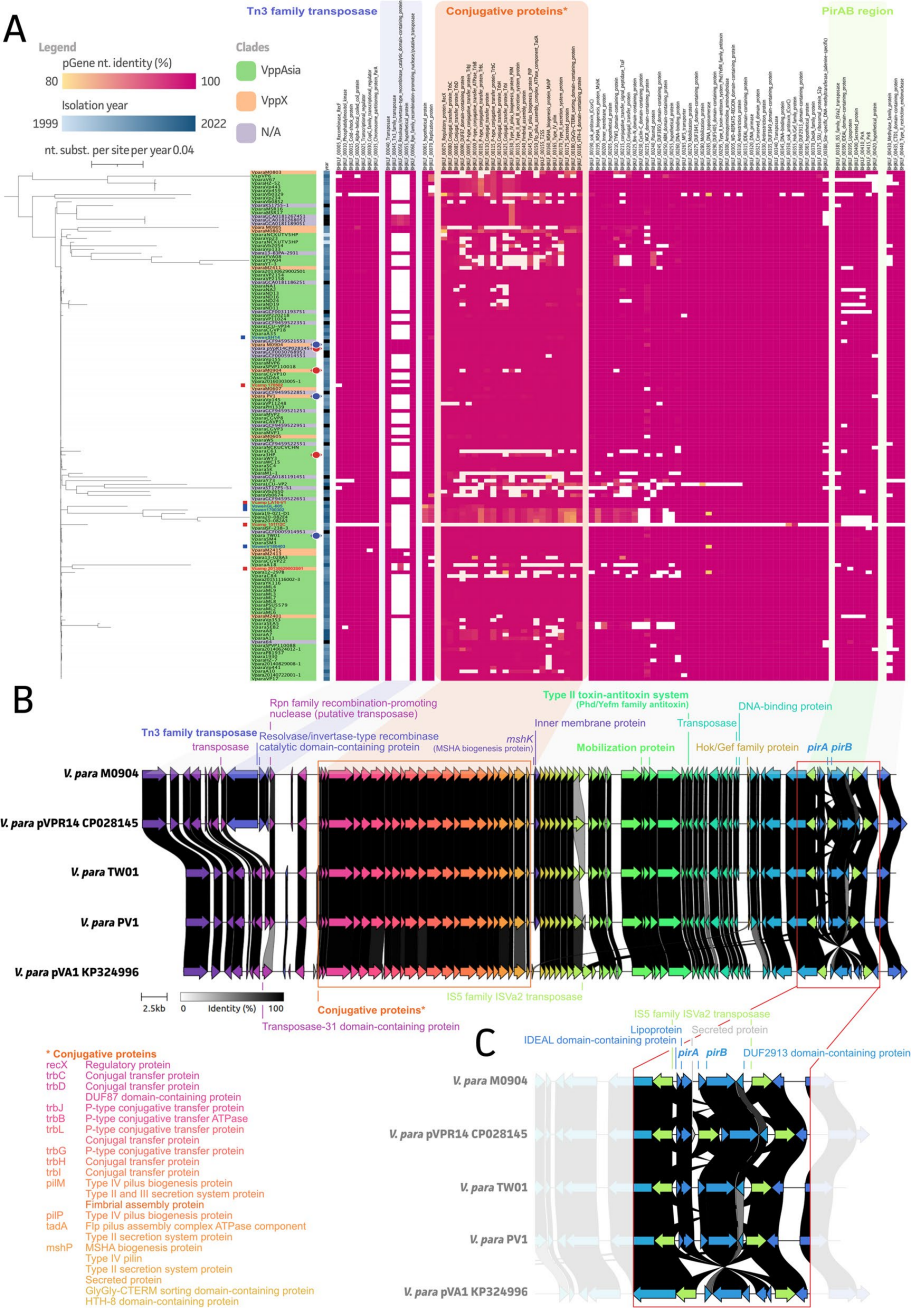
Maximum-Likelihood (ML) tree of pirAB-associated *V. parahaemolyticus* plasmid sequences and identified plasmid genes (pgenes). **A** All identified genes are classified per region in the plasmid based on the pVPR14 (CP028145). Our three new strains and the reference strains were indicated with blue and red circles, respectively and clades were colored using the color code presented by Yang et al., 2019 [21]. Plasmids originating from *V. campbellii* and *V. owensii* isolates were indicated with a red and blue box, respectively; **B** The genetic landscape of our three new isolates in relation to some reference strains; **C** Zoom-in on the versatile pirAB region within the plasmid highlighting

With a focus on AHPND, all available *Vibrio* spp. genomes from NCBI (24,758 accessed on 05/24/2023) were screened for the presence of *pirA* and *pirB* genes. A total of 142 (0.6%) of all available genome assemblies were *pirA* and/or *pirB* positive, including 133 *V. parahaemolyticus*, four *V. campbellii*, four *V. owensii*, and one *Vibrio* sp. A phylogenetic inference of these plasmids, encoding AHPND-associated genes, showed a highly conserved plasmid backbone (Fig. 1A and Additional file 1: Table S6). The region encoding for a Tn3 family transposase was the most variable one, showing its presence in 30 (21.1%) of the *pirAB*-associated plasmids. While ten (out of a total of 13 VppX strains; 76.9%) of these belonged to the VppX cluster (orange), five (out of a total of 108 VppAsia strains; 4.6%) belonged to the VppAsia cluster (green). For the remaining 15 (out of 21; 71.4%) no data on strain location was available. The plasmids originating from other *Vibrio* species clustered along the Maximum-Likelihood (ML) tree and did not show diverging plasmid compositions, except for strain 15112C from *V. campbellii*, which only had a few matching plasmid genes. Our two Asian strains (TW01 and PV1) and the M0904 Mexican strain showed high sequence concordance and plasmid build-up as compared to the used reference (pVPR14; CP028145) (Fig. 1B). As highlighted in Fig. 1C, our M0904 strain showed the presence of a Tn3 family transposon comparable to the pVPR14 (CP028145) reference plasmid. This transposon was missing in both Asian strains as complying with the pVA1 (KP324996) reference plasmid. While the conjugative protein operons, mobilization proteins, and the type II toxin-antitoxin system were highly conserved between all plasmids, the PirA/B region showed some heterogeneity (Fig. 1C). An extra IS5 family ISVa2 transposon was found in the pVPR14 (CP028145) plasmid immediately upstream of the *pirA* coding sequence, which was absent in our M0904 strain. This PirA/B region was conserved in our TW01 and PV1 strain, whereas the pVA1 (KP324996) plasmid showed an inverted PirA/B region between the two IS5 family ISVa2 transposons.

### Prevalence and genome-wide associations of common virulence-associated genes in *V. parahaemolyticus*

**Fig. 2:**
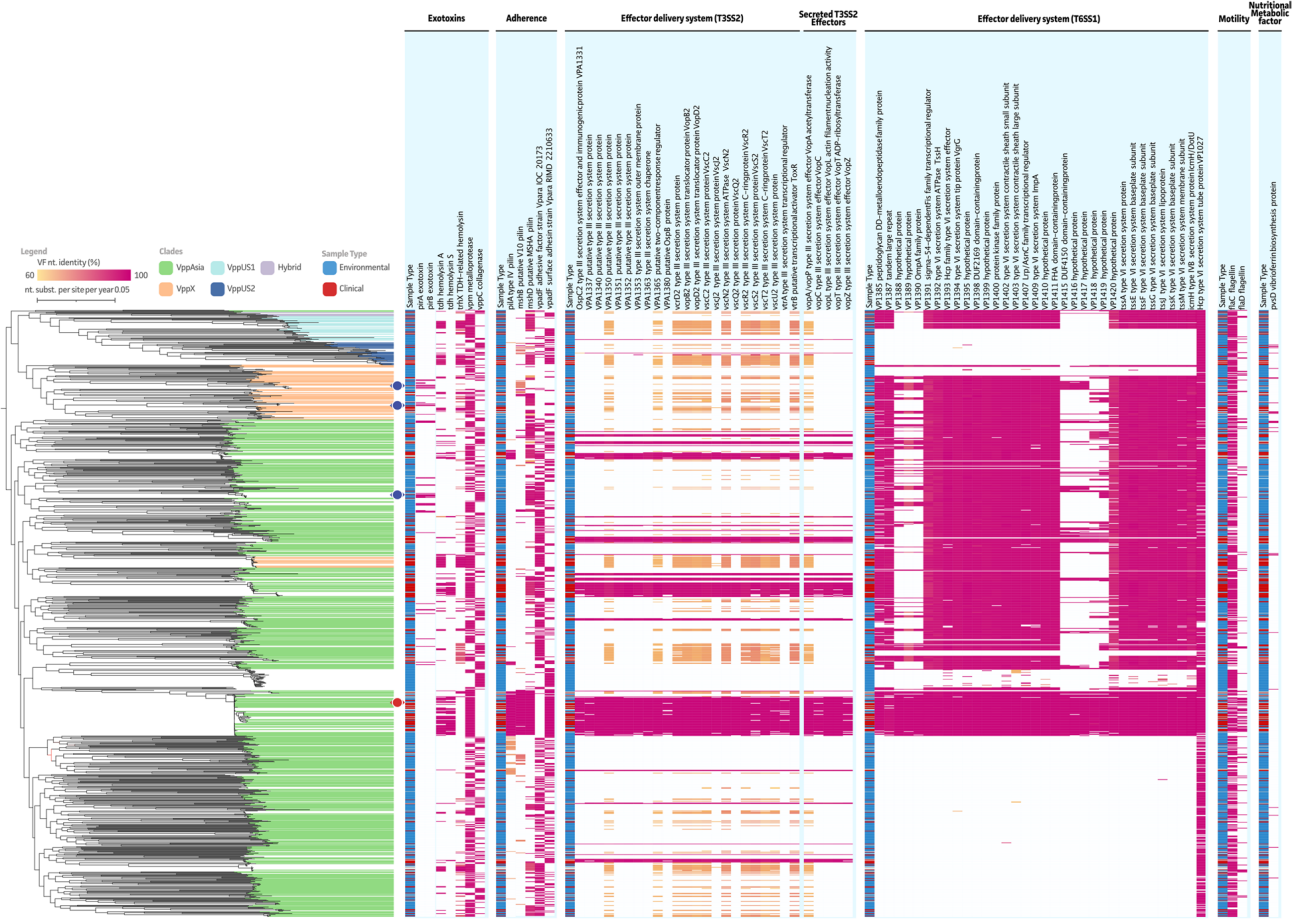
Maximum-Likelihood (ML) tree of subsampled *V. parahaemolyticus* WGS assemblies and identified virulence factor (VF) genes. All identified VFs are classified per subcategory and clades were colored using the color code presented by Yang et al., 2019 [21]. A repeated metadata band is represented highlighting the clinical (red) or environmental (blue) type of the samples. Our three new strains and the reference strain (*V. parahaemolyticus* RIMD 2210633; NC_004603 and NC_004605) were indicated with blue and red circles, respectively

From a total of 301 putative virulence factors, 224 were shown to be present in 90% of our isolates (Additional file 1: Table S5). Hence, these were excluded from further analyses. The remaining 77 genes were visualized in Fig. 2. A relatively low prevalence of *pirA* (4.8%) and *pirB* (4.3%) was observed, with 10% of the *pirA* + genotypes showing the presence of *pirA* only (*i.e.,* complete *pirB* deletion or partial below Abricate cut-offs; –minid 80 –mincov 80). While our three newly sequenced strains were all *pirA*/*pirB* positive, they lacked the chromosome-encoded *tdh* and *trh* genes. The overall prevalence of the TDH hemolysin and TRH hemolysin were estimated at 18.9% and 19.3%, respectively. The presence of the TDH hemolysin showed an apparent correlation with the presence of genes linked to the Type III Secretion System on its second chromosome (T3SS2 on NC_004605 as reference). These genes were found to be present in the *V. parahaemolyticus* population ranging between 13.7% and 15.7%. Of note, for some T3SS2 genes, other *V. parahaemolyticus* strains showed the presence of T3SS2 genes at a lower nucleotide identity, raising its prevalence to 34.1%-36.8% (orange-yellow in Fig. 2). Interestingly, when looking at the Type VI Secretion System on the first chromosome (T6SS1 on NC_004603 as reference), a clear clade-dependent trend can be observed. This results in a clear split within the VppAsia cluster (green), which is also true for the VppX (beige) and VppUS1 (light blue) clusters. The VppUS2 (dark blue) strains do not seem to possess the T6SS. Overall the presence of T6SS-associated genes was estimated to range between 56.5% and 58.6%. Comparable to the exclusiveness of *pirA*/*pirB* or *tdh*/*trh* genes within a single *V. parahaemolyticus* genome, exotoxin genes *vpm* (a metalloprotease with an estimated prevalence of 64.5%) and *vppC* (a collagenase with an estimated prevalence of 33.7%) were shown to never occur together in any of the studied strains. Furthermore, only 1.8% of *V. parahaemolyticus* are suggested to be negative for both exotoxins, the genotype to which our new *V. parahaemolyticus* PV1 strain also belongs. To completion, virulence factors involved in adherence, motility, and nutritional metabolism were included (Fig. 2 and Additional file 1: Table S5).

To understand the involvement of other genetic mediators in both AHPND and human acute gastroenteritis phenotypes, a gene-based genome-wide association study (GWAS) was performed for the *pirAB* and *tdh/trh* genes. The top 75 associated genes for *pirA* and *tdh* are highlighted in Table 2, whereas the results for *pirB* and *trh* are enclosed as Additional file 1: Table S8 and S10. Of note, for *pirAB* GWAS outputs, the *pirAB*-plasmid associated genes were filtered out, as it is evident that a plasmid-encoded gene will be highly associated with other plasmid-located genes. A complete overview can be found in Additional file 1: Table S7-S10. From our 75 top genes associated with *pirA* presence, 23 were proteins with predicted domains, a domain of unknown function (DUF), or hypothetical proteins. The remaining 52 non-*pirA* plasmid associated genes comprised proteins belonging to the family of transposable elements (*n* = 8), outer membrane proteins and fimbriae (*n* = 9, including ompA-like and OMP-b-brl proteins), metabolic enzymes (*n* = 6, including Glycosyltransferase, maltose ABC transporter permease *MalF*, maltose/maltodextrin ABC transporter substrate-binding protein *MalE*, Acyl-CoA hydrolase, and Glucose-6-phosphate isomerase) phage-associated proteins (*n* = 4), recombinases (*n* = 3), restriction endonucleases (*n* = 2), transcriptional regulators (*n* = 2), Immunoglobulin-like proteins (*n* = 2), the YjbH adaptor protein, the toxin *ccdB*, the endoribonuclease *mazF*, a lipoprotein, a Spy/CpxP family protein refolding chaperone, and methyltransferase. A GWAS analysis for *pirB* resulted in the same set of associated genes (Additional file 1: Table S8), with the addition of some extra associated genes (maltose/maltodextrin ABC transporter substrate-binding protein MalE, a holin (UPI0001BC6B02), Lipoprotein GfcB, amongst other proteins with predicted/DUF domains). Of note, these genes were also found in the *pirA* non-plasmid associated top 100 list (indicated in red in Additional file 1: Table S7 and S8).

**Table 2:**
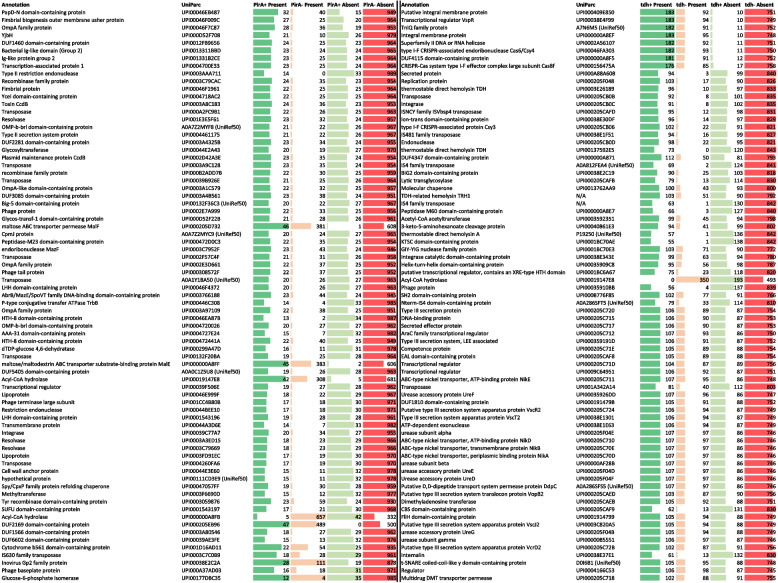
Top 75 associated genes with *pirA* and *tdh* presence. Gene annotations are shown along with their UniParc accession where available. For some genes, only a UniRef accession was available. Bars indicated number of isolates present/absent in positive (*pirA +* or *tdh +*) or negative (*pirA-* or *tdh-*) strains. For *pirA*, the top 75 non-*pirA* plasmid-associated genes are highlighted

For the *tdh* gene, 18 of the top 75 associated genes were proteins with putative functions, a predicted domain, or DUF. Interestingly, the GWAS confirms the association of Type III secretion genes (*n* = 7, including *vcrD2*, *vopB2*, *vscJ2*, *vscR2*, and *vscT2*) with the presence of the *tdh* gene. Next to this also urease-associated genes (*n* = 7, including urease subunit alpha, beta, and gamma, along with accessory genes *ureDEFG*), transcriptional regulators (*n* = 6, including *vspR*), transposable elements (*n* = 6), metabolic enzymes (*n* = 4, including acetyl-CoA acetyltransferase, 3-keto-5-aminohexanoate cleavage protein, acyl-CoA hydrolase, dimethyl adenosine transferase), an ABC-type nickel transporter operon (*n* = 4, *nikABDE*), a type I-F CRISPR-associated endoribonuclease Cas6/Csy4 and CRISPR-Cas system type I-F effector complex large subunit Cas8f, an uncharacterized secreted (effector) protein (UPI000A88A608 and UPI000205C717), a lytic trans glycosylase, a competence protein, an internalin, and a multidrug DMT transporter permease were found to be associated with *tdh* positive strains. Again, comparable results were obtained for the GWAS using *trh* positivity. Still some extra genes popped up, including seven extra Type III secretion genes, an *OmpA*-like domain-containing protein (UPI0001914797), two transcriptional regulators, a virulence-associated protein (UPI0005B6DDF1), cytotoxin (UPI000205CAF6; cytotoxic necrotizing factor Rho-activating domain-containing protein), and hemolysin B subunit protein (UPI000359204B), the Type VII secretion protein EssB, and the RTX toxin-activating lysine-acyltransferase, amongst other proteins with putative functions, a predicted domain, or DUF. Comparable to the *pirAB* GWAS outputs, most of these *trh*-associated genes were also found in the top 100 list of *tdh*-associated genes. A complete overview of the GWAS results and associated statistics is given in Additional file 1: Table S9 and S10, with extra *trh*-associated genes indicated in red.

## Discussion

The three strains sequenced in this study were confirmed to be of the species *Vibrio parahaemolyticus*, carrying a pVA1-like plasmid containing the AHPND-related *pirAB* genes. The genomes of these strains, along with a representative subsample of 1,036 (of a total of 8,897) *V. parahaemolyticus* genomes available on NCBI, were analyzed to strengthen our understanding of AHPND and *tdh* + /*trh* + genotypes. Furthermore, we showed that long-read ONT sequencing can deliver complete circular *V. parahaemolyticus* genome and plasmid assemblies.

The sampled genomes were screened for the presence of ARGs and other virulence genes. In general, an average of 5 ± 2 (SD) ARGs per *V. parahaemolyticus* genome could be identified. For the three newly sequenced strains, five ARGs were identified in the genomes of *Vp* TW01 and *Vp* PV1, but eight ARGs for *Vp* M0904. This was also reflected in the phenotypical antimicrobial susceptibility testing (AST), where *Vp* M0904 showed resistance to four antibiotics (out of the panel of 13), whereas *Vp* TW01 and *Vp* PV1 were only resistant against a single antibiotic drug (and one intermediately sensitive) antibiotic. As strain *Vp* M0904 is resistant to antimicrobials of three different categories (β-lactams, tetracyclines, and trimethoprim-sulfamethoxazole), it is regarded as a multidrug-resistant (MDR) organism. Our phenotypic observations against ampicillin and piperacillin could be linked with the presence of specific ARGs, namely the β-lactamases *bla*_*CARB-21*_ and *bla*_*CARB-23*_. Resistance towards tetracycline and trimethoprim-sulfamethoxazole is suggested to be due to the chromosomal presence of a *tet(B)* and *dfrA6* gene. Unfortunately, substantial phenotypic metadata is lacking for the available genomes on NCBI. However, screening the subsampled genomes revealed a high estimated prevalence of these β-lactamases in the *V. parahaemolyticus* population (54.9% and 44.4% for *bla*_*CARB-21*_ and *bla*_*CARB-23*_, respectively). Available literature also suggests a high resistance prevalence towards β-lactams, which we phenotypically confirmed in this study by the resistance of all three strains towards ampicillin and piperacillin. Larger-scale (*n* > 36) phenotypical studies are confirming this too, with high reported resistance prevalence towards both penams [*e.g.,* 45–100% to ampicillin [6, 28, 30–33] and 68–100% to penicillin [30–32]] and cephalosporins [*e.g.,* 49.6–84% to cefazolin [31, 34], 52–73% to cefotaxime [33, 35] and 24–82% to cephalothin [30, 34]].

An estimated 3.1% of strains showed the presence of the *tet(B)* gene, with a lower prevalence of other tetracycline resistance-associated genes such as *tet(59)*, *tet(A)*, *tet(C)*, *tet(E)*, *tetM*. Comparable results were obtained for *dfrA6* prevalence (4.0%), with the rare identification of *dfrA1*, *dfrA16*, *dfrA23*, *dfrA27*, and *dfrA32* potentially conferring strains resistant to trimethoprim-sulfamethoxazole. In addition, even though no phenotypic resistance was observed in our strains, ARG prevalence suggests low prevalence of resistance towards aminoglycosides (aminoglycoside acetyltransferase, nucleotidyltransferase, and phosphotransferase genes) and fluoroquinolones (quinolone resistance genes). This is again reflected in phenotypical studies on *V. parahaemolyticus* clinical and environmental strains, where low prevalence of resistance towards tetracyclines [*e.g,.* 0.3% to doxycycline [30, 31, 36], 0–15% to tetracycline [27, 28, 30–33, 35, 36]], trimethoprim-sulfamethoxazole [0–7% [27, 30, 31, 33]], and fluoroquinolones (*e.g,.* 0% to ciprofloxacin [27, 30] and 0–1% to levofloxacin [27, 33]] were reported. However, reported resistance prevalence against aminoglycosides is very variable [*e.g,.* 0–64% to amikacin [27, 28, 30, 33] and 15–50% to kanamycin [30, 33, 34]]. It should be noted that resistance to aminoglycosides is often the result of mutations of the ribosome [37], a factor that was not included in our analysis. As a result, there is a possibility of underestimating the extent of this resistance. Furthermore, most of the phenotypical AST screening studies focused on samples from one geographical region, and there might be large differences between *V. parahaemolyticus* populations of different locations. Furthermore, differences in applied AST screening methods (*i.e.,* disc diffusion testing v*ersus* broth microdilution method), as well as sample types (*e.g.,* clinical, environmental, seafood or surface water samples, among others), differ between studies, possibly resulting in large differences and potential underestimations of actual resistance prevalence towards certain antibiotics. Hence, using WGS-based ARG genotyping in *V. parahaemolyticus* seems to be a good predictive tool to identify, study, understand, and follow up on the dissemination of drug-resistance genotypes in the population. Many studies reported the presence of at least one multidrug-resistant isolate like *Vp* M0904 [27, 28, 38]. These isolates represent an even bigger threat to aquaculture and human health, as they are more difficult to control with antibiotics, and are an important reservoir of ARGs. This is also highlighted in the identification of a *V. parahaemolyticus* cluster showing the presence of a putative plasmid carrying multiple ARGs. Most of the genomes presented within this genetic clade originate from China and were part of an unpublished study on the occurrence and genetic environments of *bla*_*CTX-M-14*_ among foodborne *Vibrio* spp. (PRJNA622672). Still, researchers should be encouraged to link phenotypic and genotypic observations and be aware of potential ARGs not contributing to resistance phenotypes and/or being the result of contaminating contigs in the assembly. To completion, point mutations (*e.g.,* in rRNA, *gyrA*, *parC* genes) should also be considered as a potential source of resistance, which requires accurate metadata and genomics to establish proper associations [39, 40]). This was highlighted in the very high prevalence of ARGs *CRP* (99.7%), *msbA* (99.0%), *tet(35)* (99.3%), and *ugd* (84.4%). *CRP* is suggested to be involved in macrolide, penam, and fluoroquinolone resistance [41], *msbA* in nitroimidazole resistance [42], *tet(35)* in tetracycline resistance and *ugd* in resistance against peptide antibiotics [43], but do not seem to induce resistance in *V. parahaemolyticus*. It is plausible that these are so-called ‘silent’ genes, that might only be activated in specific conditions or expressed at low levels [28, 44]. The high prevalences of especially *CRP*, *msbA*, and *tet(35)* (≥ 99%) suggests that these might be intrinsic resistance genes [45]; however, their relatively low nucleotide identity (*CRP* 80.0% ± 0.1%, *msbA* 66.5% ± 0.2%, *tet(35)* 82.6% ± 0.2%, and *ugd* 69.7% ± 0.9%) compared to other ARGs suggests that these genes might be degenerated and have lost functionality over time. In contrast, all other identified ARGs are suggested to be acquired, as these are strain-specific [45].

Next, screening of all available *Vibrio* spp. genomes showed an overall low (0.57%) prevalence of *pirA* and/or *pirB* genes, and a prevalence of 4.8% and 4.3% in *V. parahaemolyticus*, respectively. In accordance with previous research [19], the two Asian strains *Vp* TW01 and PV1 did not possess the Tn3 transposon on their plasmid, while the Latin-American (Mexican) strain *Vp* M0904 did. The *pirAB* region is usually flanked by two identical IS5 family ISVa2 transposases in opposite directions that form a composite transposon called Tn903 or Tn6264 [19]. This region was conserved in the three newly sequenced strains. As observed before [46], an extra IS5 family transposon was found in pVPR14 immediately upstream of the *pirA* coding sequence. Interestingly, this insertion seems to have no effect on the transcription of the genes, but instead inhibits translation of the PirAB proteins [46]. Furthermore, compared to the other analyzed plasmids, the *pirAB* region between the two IS5 family transposons of the pVA1 plasmid is inverted compared to the other plasmids. Here our plasmid screening and characterization showed a highly conserved backbone of the *pirAB*-encoding plasmid, highlighting the biggest genetic diversity at the Tn3 transposon region. Still, it is important to note that our plasmid analysis is limited to the available data in NCBI as insufficient sequencing depth might result in the lack of data on plasmids. Also, the choice of sequencing platform affects the actual recovery of plasmid information, hence care should be taken when performing this type of analyses on genome assemblies available in the public domain. The choice of sequencing technology might also impact the potential duplication and orientation of transposons due to their repetitive regions [47–50].

The three newly sequenced *Vp* strains, as well as the subsampled genomes, were screened for the presence of virulence-associated genes. As expected, *Vp* M0904, TW01, and PV1 possessed the *pirA* and *pirB* genes, and lacked the *tdh* and *trh* hemolysin genes. Furthermore, the presence of *tdh* was strongly associated with the presence of genes linked to the T3SS2. These observations confirm the suggestions made before, that: (i) AHPND genotypes are not pathogenic to humans since they lack the *tdh* and *trh* genes, along with the absence of the T3SS2 and (ii) there is a correlation between the presence of *tdh* and *trh* genes and the T3SS2 [22, 23]. Genes related to the T6SS on the first chromosome (T6SS1) were absent in the VppUS2 clade, and present in only a subpart of the other three clades VppAsia, VppX, and VppUS1. Although previously it has been suggested that T6SS1 is present in all AHPND and human clinical isolated, and not in non-pathogenic environmental strains [22], this is not supported by our results.

Many *Vp* AHPND strains have been identified and different rates of mortality in infected shrimps were reported [51], indicating that the PirAB toxin is not the only factor contributing to virulence in shrimp. The same holds for gastroenteritis in humans, where illness is linked with the presence of *tdh/trh* and T3SS2, but other factors might contribute to virulence. To gain more insight into contributing factors, a GWAS was performed for the *pirAB* and *tdh/trh* genes. For *pirAB* it is important to note that most associations were with the plasmid genes themselves, as it is evident that a plasmid-encoded gene will be highly associated with other plasmid-located genes. These genes have been filtered out in our analyses so we could focus better on chromosome-encoded associations. Many transposon- (*n* = 8), phage-related (*n* = 4) proteins were identified. Both are often associated with the insertion of new genes primarily by horizontal gene transfer and expressing novel pathogenic properties [51], and CRISPRs are suggested to be able to prevent the invasion of prophages into bacteria, being a barrier of defense to fight against foreign DNA [51]. A previous study indicated the lack of these CRISPR elements in *Vp* AHPND strains, allowing the prophage insertion of virulence genes, possibly contributing to the virulence of *Vp* AHPND strains [51]. These observations seem to be confirmed in the present GWAS. Multiple outer membrane proteins (OMPs) were also among the associated genes, of which several were related to OmpA. For many Gram-negative bacteria, this is a key virulence factor involved in bacterial biofilm formation, eukaryotic cell infection, antibiotic resistance, and immunomodulation [52]. In *V. parahaemolyticus*, several OMPs have been shown to be involved in ampicillin resistance [53] and osmoregulation [54]. Furthermore, a Spy/CpxP family protein refolding chaperone was present in the GWAS of *pirAB*, and is demonstrated to protect OMPs against protein-unfolding stress in *E. coli* [55]. Possibly, it is important in the protection against unfolding of the previously mentioned OmpA and other OMPs. The association with two immunoglobulin-like domain-containing proteins is seen, which are often surface proteins involved in cell-to-cell recognition, adhesion, biofilm formation, and conjugative transfer [56, 57]. These domains are widely present in numerous proteins, and several have been identified in *V. parahaemolyticus*, such as adhesive factor VpadF [56, 57]. Association with toxin gene *ccdB* indicated the importance of the toxin-antitoxin (TA) system ccdA-ccdB, consisting of the stable toxin ccdB and less stable antitoxin ccdA to neutralize the toxin. It is defined as a plasmid maintenance system, acting through post-segregational killing of plasmid-free cells [58, 59]. The endoribonuclease *mazF* is also part of the chromosomal type II TA system *mazEF*. It has been identified in multiple bacteria where it regulates virulence factors, such as biofilm production in *E. coli* and in *Staphylococcus* (*S.*) *aureus*, but it may be involved in other functions such as antibiotic resistance and programmed cell death under stressful conditions [59, 60]. However, its role in *Vibrionaceae* has not been studied to the author’s knowledge.

The GWAS of the *tdh/trh* genes, related to human gastroenteritis, confirmed the association with the T3SS2, as seven genes were present in the top 75. Additionally, multiple urease-associated genes are linked to *tdh*, which are virulence factors in various pathogenic bacteria [61]. The ability of ureases to raise the pH in the immediate environment inside the host contributes to the survival of the bacteria in the host digestive tract [38]. Although there seems to be an association between *tdh* and urease genes, phenotypically this association seems to be more correct for *trh* since multiple reports have mentioned that all *trh* genotypes were urease positive, which was not the case for *tdh* [62, 63]. The association with *trh* was also confirmed in our GWAS, and the urease-related genes were ranked higher in the top 75 than for *tdh* (7 genes ranked between place 27 and 41 for *trh* compared to 7 genes ranked between place 51 and 70 for *tdh*). Furthermore, it was demonstrated that the genes for an ATP-binding cassette-type nickel transport system, which may play a role in nickel transport through the bacterial cytoplasmic membrane, are located adjacent to the urease gene cluster on the genome of *V. parahaemolyticus*, and that this region is in close proximity to the *trh* gene [63, 64]. Interestingly, this nickel transport system is indeed associated with *tdh* and *trh* according to the current GWAS. Possibly, this system aids in providing nickel for incorporation into the metallocentre within the active sites of the ureases [63]. Another interesting finding from the *tdh*-based GWAS was the association of a type I-F CRISPR-associated endoribonuclease Cas6/Csy4. This is related to a variant of the subtype I-F CRISPR-Cas system called the ‘minimal’ type, which showed a strong association with *tdh* in previous reports as well [65, 66]. Furthermore, genes for TniQ and a Cas8f-like protein also returned in our GWAS, which are all possibly linked with the minimal type I-F system. Interestingly, it was shown that this system was present within a Tn7-like transposon in *V. parahaemolyticus* and that the pathogenicity island containing the T3SS2 was correlated [67]. Association with *trh* on the other hand with genes of this minimal type I-F CRISPR-Cas system was a lot lower. Furthermore, the presence of many transposable elements (*n* = 6) as well as a competence protein is underlining the importance of genetic diversity that is associated with these genes.

## Conclusion

This study has provided a comprehensive characterization of three AHPND-inducing *V. parahaemolyticus* strains and their associated PirAB-encoding plasmids. Additionally, to the author’s knowledge, this is the first in-depth and comprehensive genome mining study on *V. parahaemolyticus* ARGs and virulence genes. The presence of ARGs and important virulence genes were examined and correlated with phylogenomic analysis of an extensive dataset of *V. parahaemolyticus* genomes. We recommend this approach to fellow researchers for rapid antibiotic resistance screening and to contain dissemination of the ARGs and resistance plasmids, particularly in light of the emergence of multidrug-resistant strains, as observed not only in this study but also in related studies. Furthermore, through GWAS, we have successfully identified known and novel genetic markers associated with the AHPND and *tdh* + */trh* + genotypes of *V. parahaemolyticus*. This discovery holds promise for the development of rapid and precise discriminatory tests targeting these genotypic variations. Collectively, these findings significantly enhance our comprehension of pathogenic *V. parahaemolyticus*, underscoring the importance of close monitoring of antimicrobial resistance of the bacteria.

## Methods

### Bacterial strains and growth conditions

Three new *V. parahaemolyticus* strains were used in this study: M0904, isolated in northwestern Mexico (originally received from A.C. Mazatlàn unit of Aquaculture), TW01, isolated in Southern Thailand, and PV1, isolated in China (both received from Robins McIntosh). All three strains were isolated from AHPND-affected shrimp and were previously confirmed to produce PirAB by Western Blot [68]. One colony of each strain was inoculated in Marine Broth (MB) (Carl Roth – CP73.1) and grown overnight (6 ± 2 h) at 28°C with shaking at 140 rpm.

### Antibiotic susceptibility testing

The three *V. parahaemolyticus* strains and the reference strain *Escherichia* (*E.*) *coli* ATCC 25922 (strain LMG8223 from the Belgian Coordinated Collection of Microorganisms BCCM) were tested for antibiotic resistance using the disc diffusion method, in compliance with the Clinical and Laboratory Standards Institute (CLSI) M45 guidelines for *Vibrio* spp. A total of 13 different antibiotics (all purchased from Oxoid Limited, UK) were tested: ampicillin (10 µg), amoxicillin-clavulanate (20/10 µg), ampicillin-sulbactam (10/10), piperacillin (100 µg), cefotaxime (30 µg), ceftazidime (30 µg), amikacin (30 µg), gentamicin (10 µg), tetracycline (30 µg), ciprofloxacin (5 µg), levofloxacin (5 µg), ofloxacin (5 µg) and trimethoprim-sulfamethoxazole (1.25/23.75 µg). Briefly, the bacteria were grown on Mueller–Hinton Agar (MHA, Carl Roth, Germany) plates after which a direct colony suspension was prepared in a 0.85% NaCl solution, and turbidity was adjusted to 0.5 McFarland standard. The MHA plates were inoculated with 100 µL of this suspension which was spread with a sterile triangle rod. Plates were allowed to dry for 5 to 10 min prior to applying the antibiotic disks onto the agar with sterile tweezers. The plates were incubated inverted at 35°C for 16 ± 2 h. The reference strain *E. coli* ATCC 25922 was used as a control to monitor the accuracy of the disk diffusion tests.

### Genomic DNA extraction and whole genome long-read sequencing

A freshly grown overnight culture of each of the three strains was subjected to the isolation of High Molecular Weight (HMW) DNA using the DNA MiniPrep Kit (Zymo Research, USA) as described before [40]. Manufacturer’s instructions were followed with the addition of a 30-min proteinase K (500 ng μL^−1^; Promega, USA) treatment at 56°C after two cycles of bead bashing in a TissueLyzer (30 oscillations per minute for 5 min; Qiagen, Germany). The quality of the resulting DNA was confirmed on a NanoDrop device. Whenever A_260_/A_280_ and/or A_260_/A_230_ measures did not reach 1.7 or 1.5 cut-offs, an extra DNA clean-up was performed using CleanNGS (CleanNA, The Netherlands) magnetic beads at a 1:1 ratio. For subsequent long-read Nanopore sequencing, 400 ng DNA was used in rapid library preparation (RBK-004), barcoding each sample for 48h sequencing on an R9.4.1 MinION flow cell. Data were acquired on a GridION device (Oxford Nanopore Technologies Ltd., UK), supporting real-time, super accurate base calling, and demultiplexing with guppy (v6.1.5; ONT).

### Genome assembly and annotation

The obtained reads were used in an *in-house* established bacterial genome assembly pipeline, which includes read quality checking (NanoComp v.1.10.0; [69]), read filtering (filtlong v0.2.1; –min_length 1,000 –keep_percent 95; https://github.com/rrwick/Filtlong), Trycycler subsampling (v0.5.3; –min_read_depth 50 –count 10 –genome_size 5 M; [47]) for independent genome assemblies using Flye (v2.9; –nano-hq -g 3m; [70]), raven (v1.8.1; [71]), wtdbg2 (v1.12; [72]), and miniasm_miniplish.sh (v0.3; -x ont -g 3m; https://github.com/rrwick/Minipolish) with *default* settings if not depicted. Subsequent trycycler commands were run as instructed using *default* settings, followed by read polishing using minimap2 (v.2.20; -a -x map-ont; [73]) and medaka (v.1.5.0; consensus –model r941_min_sup_g507 –batch_size 50; stitch –no-fillgaps; ONT). The quality of the resulting genomes was checked using CheckM (v.1.1.0; [74]), including 1,084 markers from 70 *Vibrio* spp. genomes. Species classification was done using rMLST via the pubMLST web interface [75]. Average Nucleotide Identity (ANI) of the three new strains against the *V. parahaemolyticus* reference strain RIMD 2210633 (NC_004605.1) was calculated using the ANI tool on the EZBioCloud web interface [76]. An overview of raw read statistics, quality checks, and NCBI accession numbers can be found in Additional file 1: Table S1.

### Screening for ARGs and virulence factor, and phylogenomic analysis

A total of 8,897 *V. parahaemolyticus* genome assemblies were available on NCBI (accessed on 24/06/2023), which were subsampled to 1,036 genomes maintaining genomic diversity with minimal loss of resolution (Additional file 1: Table S2). Subsampling was done using a dendrogram, which was generated based on genomic blast (*default* settings), containing the sequences from *V. parahaemolyticus* based on its taxonomy ID 223926. While iteratively going through the nodes of this dendrogram, the average distance of each node to its leaves was calculated. Whenever this average distance dropped below 0.01, only one representative sequence from that node was retained. All genomes, including the newly obtained sequences were subjected to a virulence factor and ARG screening using Abricate (v.1.0.1; –minid 80 –mincov 80; https://github.com/tseemann/abricate) using the pre-built CARD [77] and a custom virulence factor database, which included a more extensive list of putative *V. parahaemolyticus* virulence-associated genes (*n* = 301) based on available literature. This list can be accessed in Additional file 1: Table S3. Complete Abricate outputs can be accessed in Additional file 1: Table S4 and S5. The subsampled dataset was also subjected to a single nucleotide polymorphism (SNP) based phylogenetic analysis as described by Kaas et al*.* [78]. In short, SNPs were identified by aligning all the sequences to the reference strain *V. parahaemolyticus* RIMD 2210633 (NC_004605.1), using the nucmer aligner from the MUMmer package (v3.1, -CIlrT; [79]). From this output, a concatenated alignment of the SNPs was produced, which was subjected to maximum-likelihood (ML) phylogenetic inference using IQtree (v.1.6.1; -m GTR + I + R -nt AUTO -bb 1000; [80]) applying the GTR + F + I model and 1,000 ultrafast bootstraps (–ufboot). Final tree visualizations were done in iTOL (v.5; [81]).

### Plasmid characterization and genome-wide association study

All *pirA* and/or *pirB* positive strains, as obtained by virulence factor screening, were analyzed separately, to study their relatedness and genomic landscape. First, all *pirA* and/or *pirB* positive plasmid sequences were subjected to an SNP-based alignment as described before to obtain an ML phylogenetic inference. Next, our newly sequenced strains, along with some type strains (pVPR14: CP028145 and pVA1: KP324996) were used for plasmid annotation using Bakta (v1.8.2.; –db db-full –compliant –genus Vibrio –species parahaemolyticus –gram -; [82]). This allowed to generate a custom plasmid gene database for an Abricate-based search (–minid 80 –mincov 80) of all *pirA* and/or *pirB*-associated plasmid sequences. Database entries and Abricate output can be found in Additional file 1: Table S6. In addition, a visualization could be made of the *pirA/pirB* plasmid landscape in our new isolates. Gene maps were obtained using clinker (v.0.0.28; [83]). In addition, all subsampled genomes and new isolates were subjected to whole genome annotation using Bakta, with identical settings as depicted before, for use in a genome-wide association study (GWAS). The latter was done using Roary (v.3.13.0; -p 8 -e -n -v; [84]) and Scoary (v.3.6.16; -e 1000 -n tree.newick; [85]), allowing the association of coding sequences based on any pheno- or genotypic feature. Here, *pirA/pirB*/*tdh*/*trh* presence (addressed 1) and absence (addressed 0) were used in the GWAS metadata file as obtained from the Abricate output and highlighted in green in Additional file 1: Table S5. A complete overview of GWAS outputs can be found in Additional file 1: Table S7-S10.

### Supplementary Information

**Additional file 1.** 

** Additional file 2.**

## Data Availability

The draft sequences of all three *Vibrio parahaemolyticus* strains can be found on NCBI (*Vp* M0904 CP133891-CP133899, *Vp* PV1 JAVKPG000000000, *Vp* TW01 CP133900-CP133905).
