# Supplementary material for: Genomic mining of Vibrio parahaemolyticus highlights prevalence of antimicrobial resistance genes and new genetic markers associated with AHPND and tdh + /trh + genotypes
Source: BMC Genomics. 2024 Feb 14;25:178. doi: 10.1186/s12864-024-10093-9 (PMC10868097; doi:10.1186/s12864-024-10093-9)
Supplement: Supplementary file 2 — Additional file 2. [file 12864_2024_10093_MOESM2_ESM.docx]

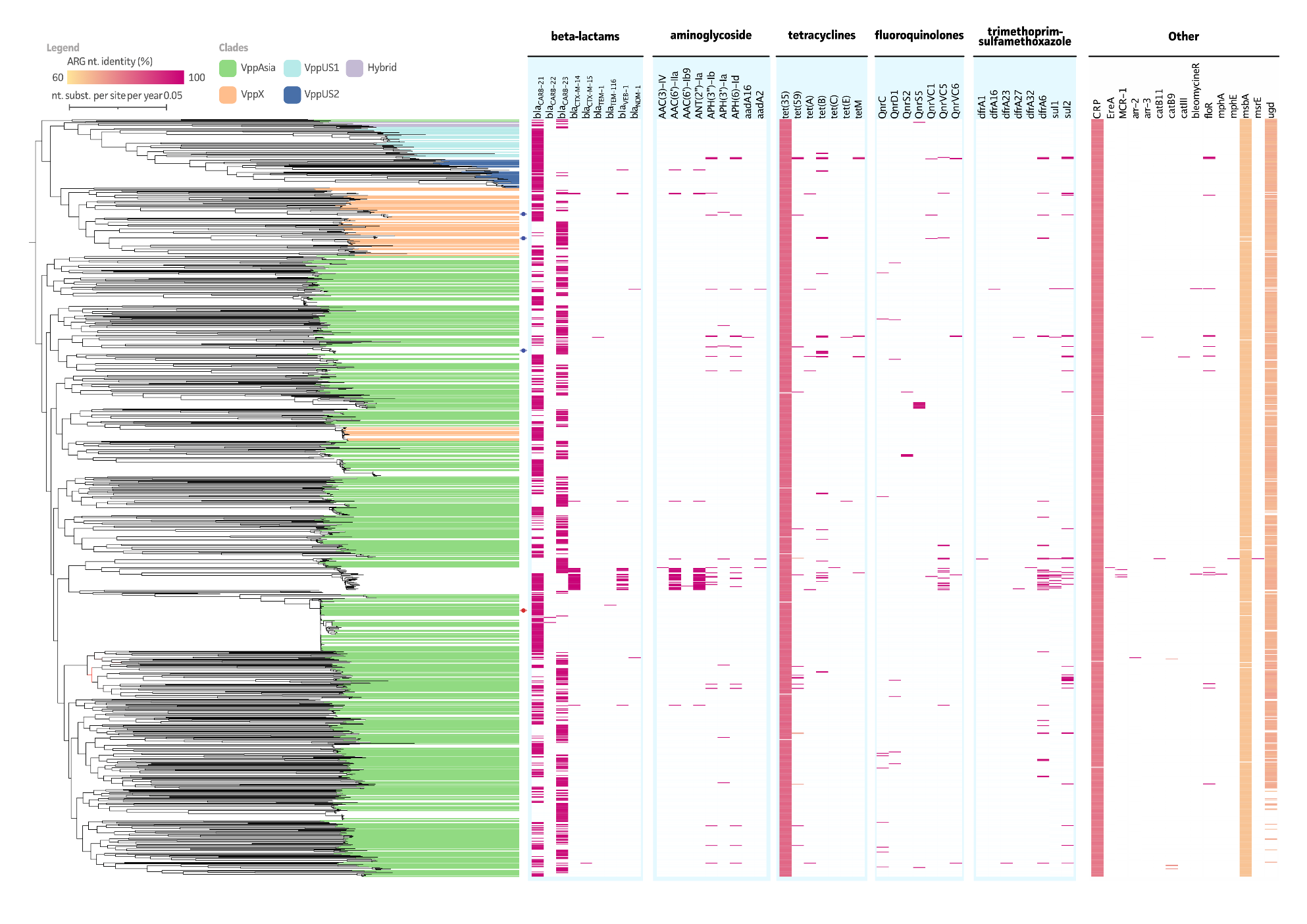


**Additional file 2: Maximum-Likelihood (ML) tree of subsampled V parahaemolyticus WGS assemblies and identified antimicrobial resistance genes (ARGs)**. All identified ARGs are classified per antimicrobial drug category and clades were colored using the color-code presented by Yang *et al.,* 2019 (21). Our three new strains and the reference strain (*V. parahaemolyticus* RIMD 2210633; NC_004603 and NC_004605) were indicated with blue and red circles, respectively.
